# Antimicrobial Effects of Essential Oils on Oral Microbiota Biofilms: The Toothbrush In Vitro Model

**DOI:** 10.3390/antibiotics10010021

**Published:** 2020-12-29

**Authors:** Andreia Aires, António Salvador Barreto, Teresa Semedo-Lemsaddek

**Affiliations:** 1 UCIBIO, Departamento de Ciências da Vida, Faculdade de Ciências e Tecnologia, Universidade Nova de Lisboa, 2829-516 Caparica, Portugal; as.aires@fct.unl.pt; 2 PYCC—Portuguese Yeast Culture Collection, Departamento de Ciências da Vida, Faculdade de Ciências e Tecnologia, Universidade Nova de Lisboa, 2829-516 Caparica, Portugal; 3 CIISA—Centro de Investigação Interdisciplinar em Sanidade Animal, Faculdade de Medicina Veterinária, Universidade de Lisboa, Avenida da Universidade Técnica, 1300-477 Lisboa, Portugal; asbarreto@fmv.ulisboa.pt

**Keywords:** oral microbiota, biofilm, essential oils, antimicrobial activity, toothbrush model

## Abstract

The present investigation intended to evaluate the bacteriostatic and bactericidal abilities of clove, oregano and thyme essential oils against oral bacteria in planktonic and biofilm states. Furthermore, aiming to mimic everyday conditions, a toothbrush in vitro model was developed. Determination of the minimum inhibitory concentration, minimum bactericidal concentration, minimum biofilm inhibitory concentration and minimum biofilm eradication concentration were achieved using the microdilution procedure. To simulate the toothbrush environment, nylon fibers were inoculated with oral bacteria, which, after incubation to allow biofilm development, were submitted to contact with the essential oils under study. Thyme and oregano essential oils revealed promising antimicrobial effects, both in growth inhibition and the destruction of cells in planktonic and biofilm states, while clove essential oil showed a weaker potential. Regarding the toothbrush in vitro model, observation of the nylon fibers under a magnifying glass proved the essential oil anti-biofilm properties. Considering the effects observed using the in vitro toothbrush model, a realistic approximation to oral biofilm establishment in an everyday use object, a putative application of essential oils as toothbrush sanitizers to help prevent the establishment of bacterial biofilm can be verified.

## 1. Introduction

The oral cavity is one of the human body settings harboring higher microbial diversity [1]. This niche presents specific conditions, such as a temperature between 35–36 °C and a constant saliva flux; thus, variations in oxygen and pH levels lead to the establishment of microhabitats, like the mucosal surfaces and the teeth [1,2]. Due to these conditions, this environment is very diverse, sheltering more than 700 bacteria, fungi, viruses and archeae [2,3]. The exact composition of the oral microbiota is very difficult to determine, but major contributors belong to the genera *Actinomyces*, *Capnocytophaga*, *Eikenella*, *Fusobacterium*, *Haemophilus*, *Leptotrichia*, *Neisseria*, *Peptostreptococcus*, *Porphyromonas*, *Prevotella*, *Propionibacterium*, *Streptococcus*, *Staphylococcus*, *Treponema* and *Veillonella* [4]. These microorganisms can also be classified as the core, similar between different individuals, or variable, in response to distinct lifestyles [5]. Due to all these variations, the necessity of maintaining a healthy microbial equilibrium is unequivocal, since an imbalance is known to lead to oral diseases [5].

In the oral cavity, the establishment of biofilms is of major relevance. Many microorganisms can form these complex structures, which result from the adherence to solid surfaces and production of extracellular polymeric substances, which protect the microbes from environmental changes, facilitate proliferation and complicate elimination [6,7]. An everyday example of an oral biofilm is the bacterial plaque formed on the teeth [8]. Consequently, regular daily hygiene will lead to the cross-contamination of toothbrushes, which will accumulate oral microbiota over time [9].

Biofilm formation can be summarized in five steps. The first describes the interaction between bacterial cells and the solid surface, resulting in the adhesion to the substrate [10]. In the second step, the microbes start producing a matrix composed mostly by polysaccharides, proteins and dead cells that act as a barrier against external perturbances [10,11]. The third step corresponds to the formation of microcolonies and the fourth to the formation of macrocolonies [10]. The last step of the biofilm formation cycle is microbial disaggregation, due to stress conditions like the lack of nutrients or space; during this stage, the cells will disperse to other areas and start the cycle all over again [10,11].

Biofilms are known to be are responsible for around 65% of the human infections in hospitals due to infiltration in medical devices, such as catheters and valves [6]. These structures are also present in the food industry and water treatment stations [6,12]. Microbes in biofilm states are a major concern, since they are much more tolerant to antibiotics and disinfectants in this conformation than in a planktonic state [6]. Oral biofilms slowly accumulate on the teeth surface, which can lead to oral diseases like cavities and gingivitis. These pathologies begin by the attachment of cariogenic species to the saliva-derived film. In this way, periodontitis starts and can escalate to periodontal disease [13]. If left untreated, it can contribute to several nonoral pathologies, such as cardiovascular diseases, diabetes or even pneumonia [14]. One of the main problems in dealing with biofilm-related infections is removing the contaminated structures, due to difficult access [8]. Another struggle is the fact that biofilms are usually polymicrobial, turning therapeutical options limited, since the antibiotics applied can be effective against some bacteria but ineffective to others [1].

Hence, it is essential to find alternatives leading to a more effective treatment, or prophylaxis, against infections/contaminations by biofilms of polymicrobial nature [6,8,14,15,16].

In the oral cavity—more specifically, in the dental pulp—there is a deposit of mesenchymal stem cells. These cells can become different types of cells and promote bone regeneration. When an aggression (inflammation or cavities) occurs, the mesenchymal stem cells can be stimulated to form osteoblast or odontoblasts, due to the signals from the surrounding environment, thus contributing to control inflammation [17,18].

Plants produce a variety of secondary metabolites that have been used from the beginning of human history for different purposes [19]. Among these are included essential oils, defined as plant’s volatile secondary metabolites responsible for a distinctive smell, taste or both [20]. These compounds can be obtained from several plant parts, such as roots, leaves or the whole plant [21]. Essential oils are complex mixtures of volatile compounds that may have demonstrated antimicrobial, antiviral, antiparasitic, antifungal and/or insecticidal activity [21]. Knowledge on the specific mechanisms by which these complex compounds act is still limited; until now, putative mechanisms have described interference with the phospholipidic bilayer, enzymatic functions or inactivation/destruction of the genetic material [22,23]. More recently, another propriety, not related with the biocidal activity of the essential oils, has been observed. These compounds demonstrated anticancer activity; more specifically, the constituents thymol and carvacrol present in some essential oils, such as oregano and [24].

Over time, a growing concern arose regarding the usage of synthetic antimicrobial agents and disinfectants once they can be prejudicial to humans and the environment, due to toxicity or the increasing of antimicrobial [25,26]. Recently, a few publications have described the risks of antiseptic resistance. For instance, it has been demonstrated that the excessive use of Cetylpiridinium Chloride (CPC), which is a monocationic quaternary ammonium compound (QAC) and a very common antiseptic used in oral care, could induce some selective pressure, leading to the development of QAC-resistant microorganisms [26,27]. In addition, the regular use of Chlorohexidine (CHX) could be connected to the appearance of a species resistant to colistin, a last resource antibiotic [28]. Therefore, one of the alternatives gaining acceptance is the use of essential oils, which present obvious advantages, like low toxicity [20]. In this context, the present study evaluated the antimicrobial effects of thyme, clove and oregano essential oils against oral bacteria in planktonic and biofilm states. Furthermore, while trying to mimic oral daily hygiene, an in vitro toothbrush model was developed to assess for essential oil putative biofilm inhibitory ability.

## 2. Materials and Methods

### 2.1. Essential Oils

The essential oils used in this study (obtained from *Thymus vulgaris* L., *Origanum vulgare* L. and *Eugenia caryophyllata* Thunb) were supplied as commercial preparations from Soria Natural (Soria, Spain). Origin and purity were demonstrated by the quality certificates made available by the company.

### 2.2. Bacterial Strains

For this study, distinct oral bacteria were included, namely *Actinomyces viscosus* (CECT 488), *Enterococcus faecalis* (V583, OG1-10, DS16), *Streptococcus mutans*, *S. oralis*, *S. sanguinis* and *S. salivarius*. Those species were selected in order to include members of the core and variable oral microbiota, i.e., microorganisms that are common to different individuals and microorganisms that differ between individuals due to different environment stimuli, respectively [5]. Microorganisms were stored at −80 °C in Brain Heart Infusion (BHI; Scharlau, Barcelona, Spain) with 20% (*v*/*v*) glycerol and routinely grown overnight on BHI agar or broth at 37 °C.

The bacterial inocula used throughout the experimental work were prepared as described by Clinical & Laboratory Standards Institute (CLSI) [29]. Briefly, a loopful of overnight culture was suspended in 0.1 mol/L phosphate-buffered saline (PBS) in order to achieve a turbidity equivalent to a 0.5 McFarland standard diluted 1:20 in the same buffer, and the suspension obtained used for the inoculation to achieve a final microbial concentration of 1 × 10^7^ colony-forming units (CFUs)/mL.

### 2.3. Evaluation of Biofilm Production by Pure and Mixed Cultures

Biofilm production was studied using pure and mixed microbial cultures. Regarding the polymicrobial assays, distinct bacterial combinations were prepared: A—*Streptococcus mutans* + *S. oralis* + *S. sanguinis* + *S. salivarius*; B—*Streptococcus mutans* + *S. oralis* + *S. sanguinis* + *S. salivarius* + *Actinomyces viscosus*; C—*Streptococcus mutans* + *S. oralis* + *S. sanguinis* + *S. salivarius* + *Enterococcus faecalis* (OG1-10) and D—*Streptococcus mutans* + *S. oralis* + *S. sanguinis* + *S. salivarius* + *Actinomyces viscosus* + *Enterococcus faecalis* (OG1-10, DS16 and V583).

For the evaluation of the biofilm-forming ability, an adaptation of the Calgary Biofilm Device [30] was applied. To make the environment more equivalent to the oral cavity, the growth medium BHI was supplemented with 2.5 g/L of the compound mucin (Sigma-Aldrich, Germany), as previously described [31].

Briefly, BHI–mucin was distributed in 96-well polystyrene microplates, inoculated with pure or mixed bacterial suspensions (final microbial concentration of 1 × 10^7^ CFUs/mL) and incubated at 37 °C for 48 h. For each microplate, a sterility control was included (noninoculated growth medium), and all assays were performed in three independent experiments, each including triplicates. After the incubation period, the lids were retrieved, the medium discarded and the wells washed three times with 0.1-M phosphate-buffered saline (PBS). Subsequently, the incubation microplate was submitted to coloration with crystal violet, as previously described [32], to assess for biofilm production using the well bottom as the adhesion surface.

### 2.4. Evaluation of Essential Oil Antimicrobial Activity

The antimicrobial activity of clove (*Eugenia caryophyllata Thunb*), oregano (*Origanum vulgare* L.) and thyme (*Thymus vulgaris* L.) essential oils was evaluated using the microdilution method [33], followed by determination of the MIC (minimum inhibitory concentration), MBC (minimum bactericidal concentration), MBIC (minimum biofilm inhibitory concentration) and MBEC (minimum biofilm eradication concentration).

As essential oils are not soluble in water [34], they were mixed 1:1 (*v*/*v*) with suspensions of 0.15% agar (which acts as an emulsifier of the oil and promotes contact with the bacteria). Those stock solutions underwent twofold serial dilutions in BHI–mucin 96-well microplates. Subsequently, the microplates were inoculated, either with pure or mixed bacterial suspensions, and incubated for 48 h at 37 °C. Triplicate assays were performed on three separate occasions for all dilutions of the essential oils under study, and for every microplate, the following controls were added: growth control (bacteria and BHI–mucin), sterility control (noninoculated medium) and solvent control (bacteria and BHI mucin with 0.15% (*v*/*v*) agar).

The MIC was defined as the minor concentration in which no bacterial growth was observed after the incubation period (absence of visual turbidity). The bactericidal effect was determined by aseptically retrieving 5 µL of inoculum from three consecutive wells without visible turbidity, spot-inoculated onto BHI plates and incubated at 37 °C overnight. In parallel, for control purposes, 5 µL of growth control wells were also inoculated in BHI plates. After overnight incubation, the microbial growth was assessed and MBC determined as the lowest concentration of essential oil that prevented growth in BHI plates [35].

To determine the minimum biofilm inhibitory concentration, the coloration with crystal violet [36] was performed after microplate incubation. MBIC was defined as the essential oil lowest concentration that prevented biofilm formation by a comparison of the optical density values against growth control (maximum biofilm formation ability) and sterility control (absence of microbial biofilm).

Regarding the determination of the minimum biofilm eradication concentration, the process was slightly different, since 96-well microplates covered with lids with pegs (NuncTM Immunoassay Transferable Solid Phases, Thermo Fisher Scientific Inc., Waltham, Massachusetts, USA) were used [30]. Briefly, microplates were prepared with BHI–mucin (without essential oils), inoculated with pure or mixed bacterial suspensions, covered with lids harboring pegs and incubated at 37 °C for 48 h. After incubation, pegs were washed three times with PBS 0.1 M to remove nonadherent bacteria and media residues [23]. Then, the pegs were immersed in eradication microplates containing consecutive twofold dilutions of the EOs (essential oils) under study and left at room temperature for 1h. Following a washing step (three times with PBS 0.1 M), the lids were placed in new microplates containing BHI–mucin and incubated overnight at 37 °C. The biofilm eradication effect was determined by aseptically retrieving 5 µL of inoculum from three consecutive wells without visible turbidity, spot-inoculated onto BHI plates and incubated overnight at 37 °C. After incubation, the microbial growth was verified and MBEC determined as the lowest concentration of essential oil that prevented growth in BHI plates, i.e., that eradicated preformed biofilms.

### 2.5. Toothbrush In Vitro Model

One of the principal components of the fibers in a toothbrush is nylon, a thermoplastic material of the polyamide family. So, for that reason, nylon was used in the development of the toothbrush in the in vitro model. In this assay, 24-well polystyrene plates (VWR Tissue culture plates, VWR International GmbH—Darmstadt, Germany) were used. For this model, the brush head would not be suitable once the incubation would imply biofilm formation not only on the fibers but, also, on the mounting for the bristles, which does not happen in vivo because it is easier to remove with water so, if applied, it would lead to false results. Experiments were performed on three independent days, all including triplicates. For comparison purposes, a commercial mouthwash was also included.

For the toothbrush model, nylon fibers were cut to ca 1.4-cm lengths using a scalpel, placed in a flask with Ringer solution and sterilized by autoclave (121 °C, 20 min), and the absence of microbes confirmed BHI broth was then introduced on the test fibers, followed by overnight incubation at 37 °C.

For the assay, the polystyrene plates were filled with BHI–mucin and supplemented with essential oils or commercial mouthwash (water; glycerin; propylene glycol; sorbitol; poloxamer 407; cetylpyridinium chloride; potassium sorbate; sodium fluoride (total fluoride content: 225 ppm); menthol; sodium saccharin; Cl 19140; Cl42051 and aroma (>100 ppm) (carvone, *Mentha viridis* (spearmint) leaf oil, menthol and methyl salicylate) to obtain sequential twofold dilutions of the test compounds. In each well, 20 nylon fibers were aseptically placed, followed by inoculation with the mixed bacterial suspensions. The incubation proceeded for 48 h at 37 °C. A growth control (well, with BHI–mucin and without an essential oil) was always included. Afterwards, the MIC and MBC were determined, as previously explained (Section 2.4).

Regarding the antimicrobial activity against bacteria in a biofilm state, the procedure required adaptations. For MBIC determination: after incubation, the nylon fibers were retrieved with tweezers to a new 24-well plaque with 500 µL of PBS 0.1 M (washing step) and carefully observed using a magnifying glass (Nikon, Tokyo, Japan); subsequently, the buffer was substituted by 150 µL of crystal violet and incubated 15 min at room temperature; after a three times washing step with PBS 0.1 M, the crystal violet absorbed by the biofilm was solubilized using a mixture of alcohol:acetone (80:20) (*v*/*v*) and quantified by measurement of the optical density at 580 nm (SUNRISE, Tecan life sciences, Männedorf, Switzerland).

### 2.6. Data Analysis

As aforementioned, each experiment included at least three technical replicates, contained growth and sterility controls and was repeated thrice, using independent bacterial cultures prepared in distinct working days.

Each determination corresponded to the median of the ordinated results. Biofilm inhibition, or eradication, were estimated by comparison with biofilm production/bacterial growth in the absence of an antimicrobial compound.

For statistical analysis, the XL STAT (2018) was used to compare between the microorganism biofilm production (pure and in mixed cultures) and/or essential oils antimicrobial effect; for the toothbrush in vitro model, the inhibitory effect of the various essential oils was compared for each combination of essential oil/bacteria. The equivalent to the analysis of variance was achieved by the Kruskal-Wallis test with a posteriori analysis by the Conover-Iman test and by the Steel-Dwass-Critchlow-Fligner test whenever the previously mentioned one could not be applied. To compare the biofilm formation in pure and mixed cultures, the Mann-Whitney test was also performed.

## 3. Results

### 3.1. Evaluation of Biofilm Production by Pure and Mixed Cultures

**Figure 1:**
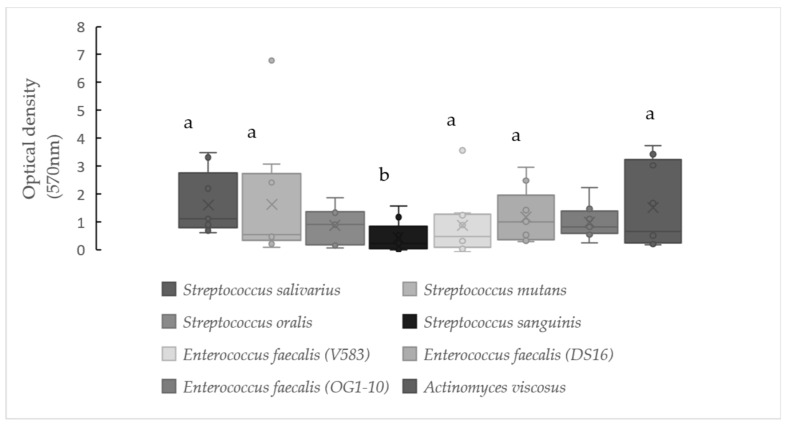
Biofilm production by pure cultures, observed after growth in Brain Hear t Infusion (BHI) with mucin at 37 °C for 48 h. Strains that do not share a common letter show a significantly different biofilm production capabilities (*p* < 0.05). The lower and upper limits of the box are the first and third quartiles, respectively, median (central horizontal bar), mean (cross) and whiskers’ upper and lower bounds (vertical lines), and outliers (point) are values that fall outside of the adjacent value region.

**Figure 2:**
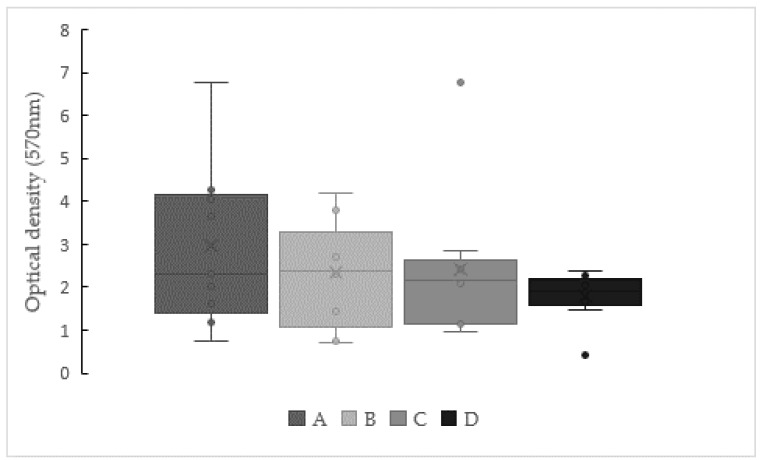
Biofilm production by mixed cultures observed after growth in BHI with mucin at 37 °C for 48 h. Legend: A—*Streptococcus mutans* + *S. oralis* + *S. sanguinis* + *S. salivarius*; B—*Streptococcus mutans* + *S. oralis* + *S. sanguinis* + *S. salivarius* + *Actinomyces viscosus*; C—*Streptococcus mutans* + *S. oralis* + *S. sanguinis* + *S. salivarius* + *Enterococcus faecalis* (OG1-10) and D—*Streptococcus mutans* + *S. oralis* + *S. sanguinis* + *S. salivarius* + *Actinomyces viscosus* + *Enterococcus faecalis* (OG1-10, DS16 and V583). No significant difference based on the Kruskal-Wallis test (*p* < 0.05). The lower and upper limits of the box are the first and third quartiles, respectively, median (central horizontal bar), mean (cross) and whiskers’ upper and lower bounds (vertical lines), and outliers (point) are values that fall outside of the adjacent value region.

Biofilm formation results for pure and mixed cultures, expressed as optical density values obtained after applying the crystal violet coloration, are displayed in Figure 1 and Figure 2. 

Regarding individual bacteria (Figure 1), the strains under study were able to produce a biofilm under the conditions analyzed, but significant differences (*p* < 0.05) were detected. Namely, *Streptococcus sanguinis* showed median optical density values around 0.2 (Q1 = 0.04 and Q3 = 0.84), while *S. salivarius, S. mutans* and *Actinomyces viscosus* presented values of approximately 0.5 to 1.1 (Q1 = 0.25–0.80 and Q3 = 2.74–3.22). *Streptococcus oralis* showed median values of t0.9 (Q1 = 0.17 and Q3 = 1.37), while the enterococci harbored 0.47 (Q1 = 0.10 and Q3 = 1.28), 1.0 (Q1 = 0.36 and Q3 = 1.95) and 0.8 (Q1 = 0.58 and Q3 = 1.38) for the strains V583, DS16 and OG1-10, respectively.

When polymicrobial suspensions were used (Figure 2), higher optical density values, i.e., a higher biofilm-forming ability, was observed. These differences were confirmed by the Mann-Whitney test (*p* < 0.05).

Analyzing mixed cultures amongst them, combination B (all streptococci + *A. viscosus*) presented a median value of maximum optical density registered at 2.4 (Q1 = 1.08 and Q3 = 3.30), while the lowest value was obtained for combination D, which included all the oral bacteria under investigation. Despite the aforementioned discrepancies, no statistical significance could be proven.

### 3.2. Evaluation of Essential Oils Antimicrobial Activity

Results obtained for each essential oil and mixed culture are displayed in Table 1.

Regarding the inhibition of planktonic bacteria (MIC), the most promising essential oil was thyme, showing median values between 3.6–7.2 mg/mL, while oregano (7.0 mg/mL) and clove (30.1–60.2 mg/mL) needed higher concentrations, revealing differences with a statistical analysis (*p* < 0.05). The combinations revealed similar sensitive behaviors to the three essential oils studied, further confirmed after applying the Kruskal-Wallis test and the Steel-Dwass-Critchlow-Fligner a posteriori test, which revealed no statistical differences between combinations (*p* < 0.05).

As expected, the minimum bactericidal concentrations were higher, between 7.2–14.4 mg/mL for thyme, 7.0–14.0 mg/mL for oregano and 30.1–60.2 mg/mL for clove. The lowest values reported for each essential oil varied amongst combinations, showing distinct bactericidal susceptibilities. The most susceptible combinations were distinct—namely, combination A and B for thyme; combination A, B and D for oregano and combination A for clove. Combination C was the most resistant to thyme and oregano and combination D for clove. Furthermore, this discrepant behavior was supported by a statistical analysis, which showed significative differences (*p* < 0.05) between thyme and oregano against clove.

Considering the inhibitory activities of the essential oils against oral bacteria in a biofilm state (MBIC and MBEC), thyme and oregano EOs showed promising antibiofilm abilities, with concentrations required for biofilm eradication, i.e., disruption of pre-formed biofilm, being higher than the necessary for the inhibition of biofilm production. As expected, bacteria in a biofilm state, despite the mixed population analyzed, were less susceptible to the essential oils than planktonic microbes. Therefore, while planktonic cells presented an inhibition concentration with median values of 3.6–7.2 mg/mL for thyme and 7.0–14.0 mg/mL for oregano, the bacteria in a biofilm state presented values in the order of 7.2–28.8 mg/mL for thyme and 14.0–28.0 mg/mL for oregano. Regarding eradication concentrations, planktonic cells presented values of 7.2–14.4 mg/mL and 7.0–14.0 mg/mL for thyme and oregano essential oils, while in a biofilm state, the values suffered an increase to 5.2–115.0 mg/mL and 14.5–111.7 mg/mL for thyme and oregano, respectively.

### 3.3. Toothbrush In Vitro Model

The present study assessed the putative antimicrobial activities of essential oils against oral bacteria. In order to complement the analysis and achieve an approximation to reality, a toothbrush in vitro model was developed. Nylon fibers were used to mimic the toothbrush, and, for comparison purposes, a commercial mouthwash was also included. Due to the expected polymicrobial nature of toothbrush bacterial contaminations, only mixed cultures were used for inoculation. The results obtained for each essential oil and mixed culture are displayed in Table 2.

Regarding minimum inhibitory concentrations, values between 11.0–15.7 mg/mL were observed for thyme and 15.7 mg/mL for oregano; no MIC could be determined for clove, since visual turbidity was observed for all tested concentrations of this essential oil. Once high concentrations of essential oils were applied, by visual observation only, it was impossible to reliably verify if the opacity corresponded to bacterial growth or to compound coloration. A statistical analysis confirmed the significant differences (*p* < 0.05) between clove essential oil and the other two compounds, thyme and oregano, by the formation of two distinct groups. These results allowed inferred that thyme and oregano essential oils are similar and more effective than clove against the bacterial combinations investigated.

When bactericidal concentrations were assessed, the tested concentrations for thyme and oregano were only effective against combination C (*Streptococcus mutans* + *S. oralis* + *S. sanguinis* + *S. salivarius* + *Enterococcus faecalis* (OG1-10)). Regarding clove essential oil, since much higher concentrations were used, a bactericidal effect was verified for all mixed cultures (values between 31.4–62.8 mg/mL). These differences were statistically validated (*p* < 0.05).

Regarding bacteria in a biofilm state, the results demonstrated the biofilm inhibition potential of the three essential oils, with thyme presenting lower MBICs. Significant differences were verified between the clove and oregano essential oils (*p* < 0.05).

Moreover, to compare the four bacterial combinations, the statistical significance of the differences observed for the MIC, MBC and MBICs was verified using the Kruskal-Wallis test, followed by the a posteriori Steel-Dwass-Critchlow-Fligner test. The results confirmed no significative differences among mixed cultures (*p* > 0.05) for all the essential oils concentrations studied.

**Figure 3:**
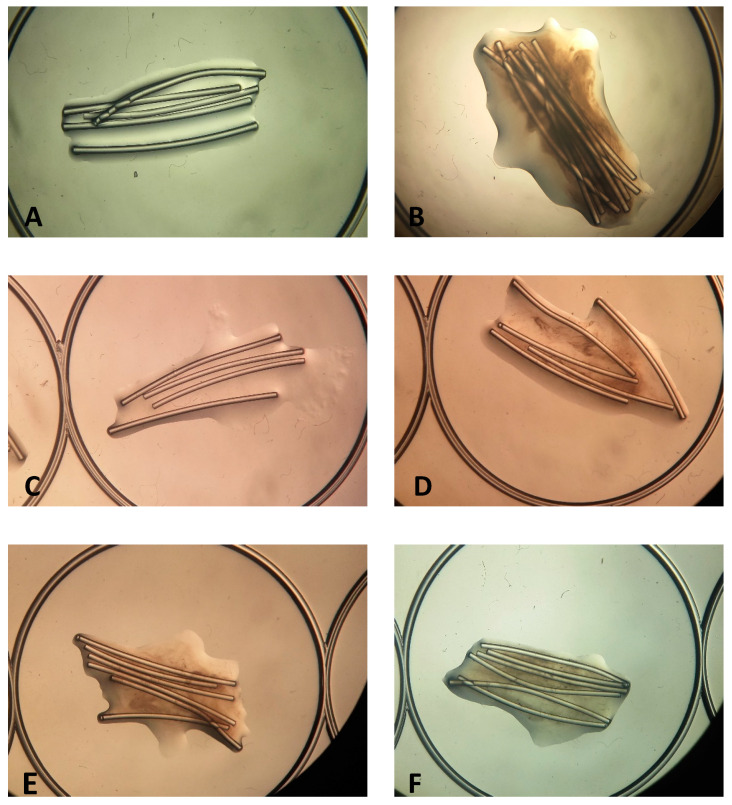
Biofilm formation in nylon fibers when in contact in different concentrations of the essential oil (EO) of oregano (observation in magnificent glass, 10× ampliation). (**A**)—Negative control, (**B**)—positive control, (**C**)—well 2 (higher concentration of EO), (**D**)—well 3, (**E**)—well 5 and (**F**)—well 6 (lower concentration of EO).

To further evaluate the antibiofilm effect, the toothbrush model was carefully observed using a magnifying glass (Figure 3). Discrepancies on the biofilm surrounding the nylon fibers are evident between conditions tested, since the biofilm increases as the essential oil concentration diminishes. Regarding the results obtained for the commercial mouthwash, an inhibition was only achieved when the compound was used in pure mode, as expected, since it corresponds to usage instructions.

## 4. Discussion

Distinct oral bacteria and bacterial combinations were evaluated regarding the biofilm-forming ability. *Actinomyces viscosus, Streptococcus oralis*, *S. sanguinis* and *S. salivarius* can be found in the oral cavity as commensals, except when an imbalance occurs, in which situation, they can be associated with oral diseases. *Streptococcus mutans* is an oral pathogen linked to oral diseases such as teeth cavities [2]. *Enterococcus faecalis*, another species included in this study, has a dual role, since it can act as commensal or as a pathogen, and this last role was associated with its presence in the oral cavity [14,22,37].

Biofilm development during growth in polystyrene microplaques, a widely used methodology [38], was applied. To approach the oral cavity environment, Brain Heart Infusion was supplemented with mucin, and the incubation temperature used corresponded to the normal human body value of 37 °C. Saliva is composed of mucins, making them abundant compounds in the oral cavity used as substrates for microbial development [37].

The comparison of biofilm production between pure and mixed cultures of oral bacteria showed an increased ability to form a biofilm when in polymicrobial mode. Hence, in nature, the existence of heterogeneous biofilms is more likely, since distinct microbes use a variety of nutrients, contributing to symbiosis and leading to a more abundant biofilm growth [39,40,41,42].

The bacterial combinations used for our study were thought to achieve a more reliable approximation to reality. Hence, the antimicrobial effect of the essential oils was only tested for bacterial combinations in order to observe how the compounds would work against multiple targets and not just against one bacterium in particular. Since the oral cavity is the ecological niche of a large variety of microbes, when an imbalance occurs, some of them will be in a higher proportion than others and may cause disease. So, using combined bacteria also mimics a more realist infection model [42].

Previous studies showed that *Streptococcus* spp. act as primary colonizers of the oral microbiota, which can maintain a stable community for large periods of time [42,43], sometimes associated with *Actinomyces* species [42,44]. Enterococci can also be present in the oral cavity as part of the biofilm but is mostly associated with infection [38,45,46].

Thyme (*Thymus vulgaris* L.), oregano (*Origanum vulgare* L.) and clove (*Eugenia caryophyllata Thunb*) are plants well-known in culinary and traditional medicine, having in common known antimicrobial proprieties [47,48,49] and usually associated with the secondary metabolites thymol, carvacrol or eugenol [47,50,51,52].

In the present study, the antimicrobial activity of thyme, oregano and clove essential oils against oral bacteria were evaluated by the microdilution method associated with the determination of the MIC (minimum inhibitory concentration), MBC (minimum bactericidal concentration), MBIC (minimum biofilm inhibitory concentration) and MBEC (minimum biofilm eradication concentration). Microbial growth was achieved approaching the oral cavity environment by using BHI supplemented with mucin for 48 h at 37 °C.

To study the activity of the essential oils against oral bacteria in both a planktonic and biofilm state, the microdilution procedure was applied, allowing the determination of the MIC, MBC, MBIC and MBEC.

The results showed a more effective inhibitory and bactericidal effect for thyme and oregano essential oils in comparison with clove. These antimicrobial activities are most likely due to the presence of the secondary metabolites thymol and carvacrol as major components [47,51], which are absent from clove essential oil [49].

These observations were further confirmed when previous studies on the effect of essential oils against oral microbiota bacteria were analyzed for comparison purposes.

Clove essential oil is commonly used as an antiseptic and analgesic to diminish tooth pain [52], since it demonstrates an antimicrobial activity against bacteria from the oral microbiota responsible for cavities and oral diseases [53,54]. Several previous studies have shown this effect against oral bacteria—namely; Aznita [55]—who studied the effect of clove in bacteria of the genera *Streptococcus*, *Lactobacillus* and *Staphylococcus* and verified that the essential oil drastically reduced the dental plaque population. Another study [56] evaluated the actions of thymol and eugenol, known to be components of thyme and clove essential oils, respectively. The effects of these secondary metabolites on the oral microbiota were evaluated, and the results showed the reduction plaque formation in the teeth and in the supragingival, as well as the reduction of gingivitis. Moreover, Timimi and Casey [57] found a significant reduction in *Streptococcus mutans* counts after the action of thyme, while Ciandrini and colleagues [58] found that carvacrol inhibits *Streptococcus mutans*, *Porphyromonas gingivalis* and *Fusobacterium nucleatum* in the planktonic and biofilm states.

More recent studies on the antimicrobial activity of essential oils focused mainly on its principal components. This approach, although important, may limit their potential, since a low amount of constituents may enhance the global outcome due to already reported synergistic effects [59].

Briefly, the work carried out by Sim et al. [60] tested the activity of oregano and thyme essential oils, as well as their major components, carvacrol and thymol, against Gram-positive and Gram-negative bacteria. These authors verified that the four components tested demonstrated good antimicrobial activity, with an inactivation period of the bacteria kill kinetic assay two times shorter than the growth control. They further demonstrated the studied compounds that presented an analogous effect, which was attributed to a similar chemical structure [60]. This study supports our observations, since the essential oils of oregano and thyme frequently presented similar outcomes.

In another study, this hypothesis of attributing the similar antimicrobial activity of thyme and oregano essential oils to the presence of the same major compound was also addressed. The action of five essential oils against Gram-positive and Gram-negative bacteria was attributed to the presence of carvacrol, justifying the light differences observed with the distinct percentages of this component in the tested essential oils [61].

Eugenol, the major compound of clove essential oil, was tested in a different study [54] that included several bacteria, among which were *E. faecalis*, *S. mutans* and *S. sanguinis*. For a long time, it has been suspected that this metabolite could be the major one responsible for the antimicrobial activity of clove. The results obtained were promising, with eugenol presenting the best antibacterial activity against oral pathogens such as *S. mutans*, (MIC of 100 µg/mL and MBC of 200 µg/mL), *S. sanguinis* (MIC of 400 µg/mL and MBC of 800 µg/mL) and *E. faecalis* (MIC and MBC of 1 µg/mL). Since this compound is absent from the thyme and oregano essential oils, this fact may be responsible for the differences between clove and the aforementioned essential oils that were observed in our investigation. In this assay, as mentioned before, the core and variable species of the oral microbiota were used. The essential oils applied showed antimicrobial effects for both, which can raise concerns by having those effects in commensal bacteria. By eradicating or inhibiting the bacteria of this group, an imbalance can occur leading to infection or oral disease, turning our solution into a problem. This way, the use of essential oils needs to be monitored.

Biofilms also have the particularity of being up to 1000 times more tolerant to antibiotics than planktonic cells [6]. This highly increased level of tolerance was not verified in the present investigation, probably due to the complex compositions of the essential oils, which resulted in a more effective biofilm penetration and access to the bacterial targets [47,49,51] that make these compounds relevant as antimicrobial alternatives. However, in a study carried out by Cieplik et al. [62], extracts of clove and oregano were tested, as well the cetylpiridinium chloride (CPC), chlorohexidine (CHX) and other compounds, against the species *S. mutans* and other two species of *Actinomyces*. Only CPC and CHX showed a reduction of ≥ 5log_10_ CFUs lower than the level of detection, while the extracts of clove and oregano showed a reduction of < 2log_10_ CFUs in the biofilm formed in 24 h, demonstrating that natural compounds are not always effective.

The period chosen of one-hour incubation with the essential oil may not be realistic when in a clinical setting, but this treatment can be applied to disinfecting the toothbrush after brushing the teeth. This setting is responsible for accumulating microorganisms once it is exposed to microbes every day and could even promote the dispersion of pathogens to noncontaminated places [9].

For this study, a toothbrush in vitro model, as aforementioned, was performed to achieve a realistic approach regarding the oral microbiota. Over time, other studies have been published discussing other realities that should be taken into consideration when performing experiments in this setting. For example, Marrelli and his colleagues conducted a study on the mechanical properties of commercially available yttrium-doped zirconia. These ceramic-based materials are used for dental restoration. In that case, strength and flexibility tests were performed, and it was observed that a polished surface will increase the strength. Besides, all ceramic-based materials showed promising potential in dental repair [63].

When planning a model-based experiment, it is important to take under consideration the morphology and geometry of the tested material. In the present study, we included the material present in the fibers of a regular toothbrush and took into account the length and shape of the nylon fibers in order to perform a model as close to reality as possible. With this approach, the obtained biofilm was expected to behave similarly to the microbial formed in real toothbrushes. In a previous report, Marrelli discussed the importance of studying the biomaterials and their interactions with the cells and the surface, as well as the use of a standardized approach to three-dimensional models. He determined that factors like the surface and shape of the material have an effect on the results, as well as the culture conditions, infantizing the importance of a normalized method [64].

In conclusion, the toothbrush in vitro model applied in the present study allowed an approximation to the microbial contamination of a daily used object. Observations proved that the efficacy of the essential oils under study point towards possible applications as biofilm inhibitors or eradicators, leading to lowering the bacterial counts in currently used household utensils.

## 5. Conclusions

The present study had, as the main objectives, the evaluation of biofilm production by pure and mixed cultures of oral bacteria, the evaluation of the antimicrobial activity of essential oils and the development of an in vitro model representing a toothbrush contaminated with oral microbiota.

Overall, the oral bacteria were able to form biofilms both in pure and mixed cultures with polymicrobial conditions, leading to an increased biofilm production. Similar behaviors were observed within members of the same genus, and no significative differences could be detected amongst the distinct bacterial combinations.

Thyme and oregano essential oils revealed promising antimicrobial effects, both in the inhibition and destruction of cells, in the planktonic and biofilm states, while clove essential oil showed a weaker potential when compared with the two compounds.

Regarding the toothbrush in vitro model, thyme and oregano also presented an effective action on the inhibition of planktonic cells and biofilm formation, while the clove essential oil showed an encouraging bactericidal effect. Observation of the nylon fibers under a magnifying glass proved these antibiofilm properties, pointing towards a putative application of essential oils as toothbrush sanitizers to help prevent the establishment of oral bacteria-related biofilms. These promising results should be communicated to endodontists, since they may lead to the establishment of toothbrush disinfection protocols and, thus, to the improvement of oral health.

## Figures and Tables

**Table 1:** Antimicrobial activity of the essential oils against mixed cultures (values displayed in mg/mL).

Mixed Cultures		Essential Oils											
		Thyme				Oregano				Clove			
	Statistics	MIC (a)	MBC (a)	MBIC (a)	MBEC (a)	MIC (a)	MBC (a)	MBIC (b)	MBEC (a)	MIC (b)	MBC (b)	MBIC (c)	MBEC (b)
**A**	Mean	7.2	9.0	12.6	45.2	7.8	9.5	18.8	58.7	30.1	41.8	37.6	322.1
	Min	3.6	3.6	1.8	0.9	3.5	3.5	3.5	14.0	15.0	15.0	15.0	3.8
	Q1	3.6	6.3	4.5	0.9	5.2	7.0	8.7	21.0	15.0	30.1	26.3	3.8
	Median	7.2	7.2	7.2	5.2	7.0	7.0	21.0	55.9	30.1	30.1	30.1	481.3
	Q3	10.8	14.4	28.8	87.1	10.5	14.0	28.0	83.8	37.6	60.2	30.1	481.3
	Max	14.4	14.4	28.8	222.8	14.0	14.0	28.0	111.7	60.2	60.2	30.1	481.3
**B**	Mean	5.2	7.2	20.4	27.1	7.8	9.8	14.8	112.9	37.0	42.6	40.1	481.3
	Min	0.4	7.2	7.2	0.9	3.5	7.0	7.0	1.7	3.8	15.0	15.0	481.3
	Q1	3.6	7.2	7.2	0.9	5.2	7.0	7.0	2.6	6.6	26.3	15.0	481.3
	Median	7.2	7.2	28.8	14.0	7.0	7.0	14.0	111.7	45.1	45.1	45.1	481.3
	Q3	7.2	7.2	28.8	27.9	10.5	14.0	24.4	167.6	60.2	60.2	60.2	481.3
	Max	7.2	7.2	28.8	111.4	14.0	14.0	28.0	446.9	60.2	60.2	60.2	481.3
**C**	Mean	11.6	16.2	28.8	21.9	12.8	16.0	22.7	46.4	55.2	45.1	51.1	361.0
	Min	3.6	3.6	28.8	0.9	3.5	7.0	7.0	0.9	15.0	30.1	15.0	240.7
	Q1	3.6	6.3	28.8	3.5	7.0	7.0	12.2	1.7	60.2	30.1	60.2	300.8
	Median	7.2	14.4	28.8	14.0	7.0	14.0	28.0	15.7	60.2	45.1	60.2	361.0
	Q3	21.6	28.8	28.8	55.7	21.0	28.0	28.0	111.7	60.2	60.2	60.2	241.4
	Max	28.8	28.8	28.8	55.7	28.0	28.0	28.0	111.7	60.2	60.2	60.2	481.3
**D**	Mean	5.2	9.6	22.2	107.0	6.6	8.4	27.9	57.0	45.1	46.8	42.1	327.0
	Min	3.6	3.6	3.6	1.8	3.5	3.5	27.9	7.2	15.0	30.1	15.0	218.0
	Q1	3.6	3.6	11.7	28.8	3.5	3.5	27.9	7.2	30.1	30.1	15.0	272.5
	Median	3.6	10.8	28.8	115.0	7.0	7.0	27.9	14.5	60.2	60.2	60.2	327.0
	Q3	7.2	14.4	28.8	230.0	7.0	14.0	27.9	101.4	60.2	60.2	60.2	381.5
	Max	7.2	14.4	28.8	230.0	14.0	14.0	27.9	231.7	60.2	60.2	60.2	436.1

Legend: A—*Streptococcus mutans* + *S. oralis* + *S. sanguinis* + *S. salivarius*; B—*Streptococcus mutans* + *S. oralis* + *S. sanguinis* + *S. salivarius* + *Actinomyces viscosus*; C—*Streptococcus mutans* + *S. oralis* + *S. sanguinis* + *S. salivarius* + *Enterococcus faecalis* (OG1-10) and D—*Streptococcus mutans* + *S. oralis* + *S. sanguinis* + *S. salivarius* + *Actinomyces viscosus* + *Enterococcus faecalis* (OG1-10, DS16 and V583). MIC—minimum inhibitory concentration. MBC—minimum bactericidal concentration. MBIC—minimum biofilm inhibitory concentration. MBEC—minimum biofilm eradication concentration. Essential oils (EOs) that do not share a common letter show a significantly different antimicrobial effect (*p* < 0.05). Each value represents the median of replicated experiments. For each combination, it is represented by the values for the first (Q1) and third (Q3) quartiles, median, mean and the maximum and minimum values.

**Table 2:** Antimicrobial activity of essential oils against mixed cultures: the toothbrush in vitro model (values displayed in mg/mL).

Mixed Cultures		Essential Oils								
		Thyme (a)			Oregano (a)			Clove (b)		
	Statistics	MIC	MBC	MBIC	MIC	MBC	MBIC	MIC	MBC	MBIC
**A**	Mean	15.3	-	2.0	12.4	-	10.3	-	62.8	11.3
	Min	13.9		2.0	7.1		2.0		62.8	7.1
	Q1	15.7		2.0	7.5		3.4		62.8	7.1
	Median	15.7		2.0	15.7		11.8		62.8	7.2
	Q3	15.7		2.0	15.7		15.7		62.8	13.7
	Max	15.7		2.0	15.7		15.7		62.8	31.4
**B**	Mean	11.4	-	2.0	14.0	-	6.6	-	62.8	8.6
	Min	7.0		2.0	7.1		2.0		62.8	7.1
	Q1	9.2		2.0	11.4		2.0		62.8	7.1
	Median	11.4		2.0	15.7		2.0		62.8	7.4
	Q3	13.5		2.0	15.7		15.7		62.8	7.9
	Max	15.7		2.0	15.7		15.7		62.8	15.7
**C**	Mean	14.9	15.7	2.0	15.4	15.7	2.0	-	62.8	16.5
	Min	14.0	15.7	2.0	14.2	15.7	2.0		62.8	7.1
	Q1	14.4	15.7	2.0	15.0	15.7	2.0		62.8	7.2
	Median	14.9	15.7	2.0	15.7	15.7	2.0		62.8	11.0
	Q3	15.3	15.7	2.0	15.7	15.7	2.0		62.8	15.7
	Max	15.7	15.7	2.0	15.7	15.7	2.0		62.8	56.9
**D**	Mean	10.9	-	2.0	15.4	-	6.6	-	31.4	18.7
	Min	7.9		2.0	14.2		2.0		31.4	7.1
	Q1	9.4		2.0	15.0		2.0		31.4	7.1
	Median	11.0		2.0	15.7		2.0		31.4	7.4
	Q3	12.5		2.0	15.7		15.7		31.4	31.4
	Max	14.0		2.0	15.7		15.7		31,4	62.8

Legend: A—*Streptococcus mutans* + *S. oralis* + *S. sanguinis* + *S. salivarius*; B—*Streptococcus mutans* + *S. oralis* + *S. sanguinis* + *S. salivarius* + *Actinomyces viscosus*; C—*Streptococcus mutans* + *S. oralis* + *S. sanguinis* + *S. salivarius* + *Enterococcus faecalis* (OG1-10) and D—*Streptococcus mutans* + *S. oralis* + *S. sanguinis* + *S. salivarius* + *Actinomyces viscosus* + *Enterococcus faecalis* (OG1-10, DS16 and V583). MIC—minimum inhibitory concentration. MBC—minimum bactericidal concentration. MBIC—minimum biofilm inhibitory concentration. EOs that do not share a common letter show significantly different antimicrobial effects (*p* < 0.05). Each value represents the median of replicated experiments. For each combination, it represents the values for the first (Q1) and third (Q3) quartiles, median, mean and the maximum and minimum values.

## Data Availability

The data presented in this study are available in the article.
